# Solvent Effect on Product Distribution in the Aerobic Autoxidation of 2-Ethylhexanal: Critical Role of Polarity

**DOI:** 10.3389/fchem.2022.855843

**Published:** 2022-03-25

**Authors:** Zheng Wang, Yitong Qin, Huijiang Huang, Guobing Li, Yan Xu, Peng Jin, Bo Peng, Yujun Zhao

**Affiliations:** ^1^ Key Laboratory for Green Chemical Technology of Ministry of Education, School of Chemical Engineering and Technology, Tianjin University, Tianjin, China; ^2^ School of Materials Science and Engineering, Hebei University of Technology, Tianjin, China; ^3^ SINOPEC Research Institute of Petroleum Processing, Beijing, China

**Keywords:** 2-ethylhexanal, oxidation, solvent effect, intermolecular forces, hydrogen bond

## Abstract

In the aerobic oxidation of aldehydes to acids, how the solvent affect the reaction remains unclear. Herein, the solvent effect in the oxidation of 2-ethylhexanal (2-ETH) to 2-ethylhexanoic acid (2-ETA) was systematically investigated. The vastly different product distributions were observed which could be ascribed to the dominant intermolecular forces. Though strong intermolecular forces in protic solvents limit the oxidation, the optimal 2-ETA yield (96%) was obtained in *i*propanol via gradually evaporating the solvent to remove the interactions. Theoretical calculations further revealed that the hydrogen bonds between reactant and protic solvent increase the C-H bond energy (-CHO in 2-ETH). Meanwhile, the hydrogen bonds may improve 2-ETA selectivity by promoting H transfer in the oxidation rearrangement step. Our work discloses the critical role of polarity in determining the reactivity and selectivity of 2-ETH oxidation, and could guide the rational design of more desirable reaction processes with solvent effect.

## 1 Introduction

Oxidation is one of the basic organic chemical reactions with many essential applications. Among them, the production of carboxylic acid via aldehyde oxidation is a widely recognized route (Guo et al., 2014; Megías-Sayago et al., 2020). For this transformation, metal salts or oxides, such as CrO_3_ (Thottathil et al., 1986), KMnO_4_ (Mahmood et al., 1999), K_2_S_2_O_8_ (Travis et al., 2003), and KIO_4_ (Hunsen, 2005) are usually used as oxidants for the reaction. However, these oxidants have a much higher environmental impact due to the large quantities of waste liquid containing heavy metal ions. With the development of green chemistry, the demand for environmental-friendly oxidants has rapidly increased. Oxygen is an ideal oxidant, as water often is the co-product during reaction. More importantly, air is easily accessible and safe for manufacturing. Hence, using air as an oxidant has attracted extensive attention (Fujiwara et al., 2020; Wang et al., 2020; Zhu et al., 2020; Wei et al., 2021).

**SCHEME 1:**
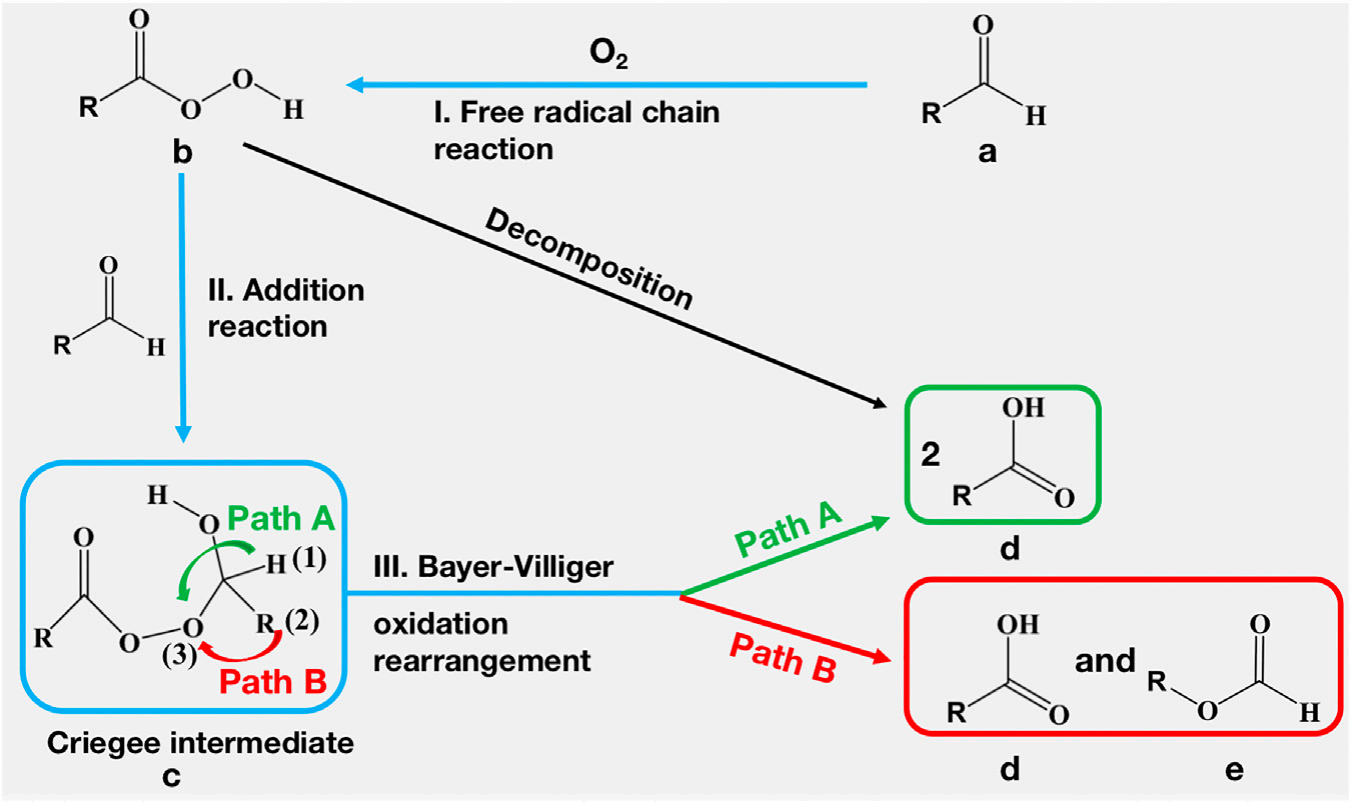
The mechanism of autoxidation reaction of aldehyde compounds.

Starting from the aldehyde compounds, the quickly occurred autoxidation under aerobic conditions is one of the most common reactions (Vanoye et al., 2013a). The widely acknowledged reaction mechanism of autoxidation is elaborated as follows (Scheme 1): I. Ground state (triplet state) oxygen first reacts with the aldehyde 1) through a free radical chain reaction to form the corresponding peroxy-acid 2); II. A Criegee intermediate 3) is formed via the nucleophilic addition reaction between peroxy-acid and aldehyde; III. The Criegee intermediate undergoes a Baeyer-Villiger (B-V) oxidative rearrangement reaction to generate the final product. In step III, after the cleavage of peroxy bond, two pathways (A & B) generate different products. In path A, two molecules of carboxylic acid 4) will be formed, while path B results in one molecule of carboxylic acid and one ester 5). Besides the reaction paths *via* a Criegee intermediate, the formation of carboxylic acid can also be achieved through the direct decomposition of the peroxy-acid (Vanoye et al., 2019). Recently, a new mechanism was proposed: after oxygen insertion, the formed acylperoxy radical will transform to peracid and carboxylic acid simultaneously (Vanoye and Favre-Reguillon, 2022).

2-ethylhexanal (2-ETH) is a typical model compound in aldehyde oxidation to produce 2-ethylhexanoic acid (2-ETA). 2-ETA is a versatile chemical with application in medicine, lubricants, cosmetics, perfumes, cold-resistant plasticizers, and many other fields. At present, there are two regular routes for producing 2-ETA, one is 2-ethylhexanol oxidation, and the other is butyraldehyde condensation-hydrogenation-oxidation. The latter process is widely used due to its high continuity and convenience of scaling-up. By comparison, there are few studies on the oxidation of 2-ETH. Under non-catalytic conditions, the selectivity of 2-ETA is ∼75% (Lehtinen et al., 2001). The oxidation rate and 2-ETA yield increased by adding catalysts and/or promoting mass transfer. Both homogeneous and heterogeneous catalysts have been reported in previous works, including Mn(II), Co(II) (Lehtinen and Brunow, 2000), Ag(I)-IPr (Liu et al., 2015), Cu(II)-SIMes (Liu and Li, 2016), Fe-based and Ag/C (Yu et al., 2017) catalysts, which can all improve the selectivity of 2-ETA. Vanoye et al. (2013b), Vanoye et al. (2014), and Vanoye et al. (2016) used a continuous flow (Taylor flow) micro-reactor made of PFA tubes and obtained higher 2-ETA selectivity (97%) with 5∼7.5 bar oxygen pressure. It is the state-of-the-art work in continuous flow reactor for aldehyde oxidation. However, several main drawbacks still hinder the current application of above process: 1) high oxygen pressure; 2) alkaline environment resulting in saline waste water; 3) difficulty of separation between homogeneous catalyst and reagents.

Solvation has been shown in many reactions to have a significant impact on the free energies of liquid reactants and surface species, and solvent molecules may also participate in the reaction as reactant or competitively adsorb to active sites (Potts et al., 2021). The particular intermolecular forces in the solvent environment will modify the reaction activity. For example, Shapiro and Vigalok (2008) and Shapiro et al. (2010) used water as the solvent, and the yield of 2-ETA reached 86% after 12 h. Lehtinen et al. (2001) applied various organic reagents to this reaction and proposed the order of reactivity of different solvents, where methanol provides the lowest reactivity. Nevertheless, the reason for the solvent effect in the reaction is still ambiguous.

In this work, we used air as the oxidant and investigated the solvent effect of the non-catalytic autoxidation of 2-ETH to 2-ETA under mild reaction conditions (35°C, 1 atm). The protic solvents gave rise to a higher selectivity of 2-ETA. Based on this, the solvent evaporation method was proposed and reaches the highest 2-ETA yield of 96%. Combining with theoretical calculations and solvent parameter equations, it is suggested that the hydrogen bonds formed between reactant and solvent enhance the H transfer in B-V reaction, thus greatly improving the 2-ETA selectivity.

## 2 Materials and Methods

### 2.1 Reagents and Materials

2-ethylhexanal (Adamas-beta, > 95%), n-hexane, acetonitrile, benzene (Tianjin Kemiou Chemical Reagent Co., Ltd. HPLC; Tianjin, China), c-hexane, methanol, ethanol, n-propanol, *i*propanol, n-butanol (Tianjin Concord Technology Co., Ltd. HPLC; Tianjin, China). All the organic solvents above are dehydrated by 3A molecular sieve (Shanghai Macklin Biochemical Co., Ltd. ; Shanghai, China) before use. Air, nitrogen (Tianjin Liufang Gas Co., Ltd. ; Tianjian, China, 99.99%).

### 2.2 Reaction Evaluation

The aerobic autoxidation reaction was carried out in a 100 ml four-neck flask. The reaction conditions are as follows: reaction temperature 35°C, reaction pressure 101.325 kPa, magnetic stirring 650 r/min, air flow rate 10 ml/min, reactant/solvent = 10 (w/w). Firstly, the solvent (30 g) was charged into the four-necked flask, then the temperature was controlled by placing the flask in an isothermal water bath with magnetic stirring of the solvent. Next, a nitrogen flow of about 50 ml/min was bubbled into the liquid in the flask to replace the air for 10 min. Finally, about 3 g reactant was added into the solvent and the gas was switched to air with a flow of 10 ml/min. The reaction was performed under reflux condensation. The products were analyzed with GC-3420A (Beijing Beifen Analytical Instruments Co., Ltd.; Beijing, China) equipped an HP-INNOWax (30 m × 250 μm × 0.5 μm) column. The calculation methods of the conversion and selectivity are as fellow (1, 2):

$$Conversion\;of\;2-ETH=\frac{Moles\;in\;feed-Moles\;in\;products}{Moles\;in\;feed}\times 100\%\;$$ (1)

$$Selectivity\;of\;2-ETA=\frac{Moles\;converted\;to\;2-ETA}{Moles\;converted\;of\;2-ETH\;}\times 100\%\;$$ (2)

### 2.3 Computational Method

The Gaussian 16 quantum chemistry software (Frisch et al., 2016) was employed for all the theoretical calculations. The calculation adopts the B3LYP (Lee et al., 1988; Becke, 1993) density functional theory (DFT) method including the D-3 dispersion correction (Grimme et al., 2010). All structure optimization and frequency analysis were performed with the 6-311G* basis set and all the reported structures are free of any imaginary frequency. The SMD implicit solvent model (Marenich et al., 2009) was used to simulate the various solvent environments (Ho et al., 2010; Ribeiro et al., 2011).

## 3 Results and Discussion

### 3.1 Product Distribution in Different Solvents

Different solvents (aprotic and protic solvents) were selected for the oxidation reaction (Table 1). For aprotic solvents, the conversion of 2-ETH (entries 1–3) is over 92%. However, the conversion shows a downward trend as the polarity of the solvent increased. When using acetonitrile (entry 4) as the solvent, the conversion of 2-ETH is only 87.2%. For protic solvents, the reaction rate decreases, and the 2-ETH conversion (entries 5–8) sharply drops below 36%. Notably, the selectivity of 2-ETA reaches the highest level (>96%) in the protic solvent. In methanol, the solvent molecular would form great interaction with the reactant and limit the 2-ETH conversion due to the strongest polarity. Lehtinen et al. (2001) performed the same oxidation reaction (methanol, O_2_, room temperature) and the 2-ETH conversion was only 8% after 2 h, which matches well with the present work.

**TABLE 1:** Product distribution after 2 h.

Entry	Solvent	Conversion/%	Selectivity/%			Yield/%
			2-ETA	i-Heptyl formate	Others	
1	n-Hexane	97.7	67.4	29.5	3.1	65.8
2	c-Hexane	92.3	69.7	29.0	1.3	64.4
3	Benzene	93.9	54.8	43.8	1.5	51.4
4	Acetonitrile	87.2	58.8	28.5	12.7	51.3
5	n-Butanol	29.5	97.4	1.8	0.8	28.7
6	*i*Propanol	35.2	96.7	1.7	1.6	34.1
7	n-Propanol	34.7	96.9	1.7	1.5	33.6
8	Ethanol	12.0	96.3	1.4	2.3	11.6
9	Methanol	—	—	—	—	—

Reaction conditions: temperature = 35°C; pressure = 101.325 kPa; magnetic stirring = 650 r/min; air flow rate = 10 ml/min; reactant/solvent = 10:1 (w/w).

In Table 2, for aprotic solvents (entries 10–13), the conversion of 2-ETH is ∼99%. The conversion remains stable with the extension of reaction time to 18 h (entries 14–18). The highest 2-ETH conversion in *i*propanol is 91.9% (entry 15), and the 2-ETA selectivity in all alcohol solvents is ∼98%. In addition, the conversion of 2-ETH under solvent-free and water (entries 19, 20) is similar to that of aprotic solvents, and the 2-ETA selectivity is ∼75%.

**TABLE 2:** Product distribution of reaction endpoint.

Entry	Solvent	Reaction time/h	Conversion/%	Selectivity/%			Yield/%
				2-ETA	i-Heptyl formate	Others	
10	n-Hexane	3	99.3	66.7	29.5	3.9	66.2
11	c-Hexane	4	98.4	74.1	24.8	1.2	72.9
12	Benzene	3	99.0	53.1	45.4	1.5	52.5
13	Acetonitrile	4	99.3	65.0	24.3	10.8	64.5
14	n-Butanol	12	81.7(82.2)^a^	98.7	1.0	0.3	81.0
15	*i*Propanol	12	91.9(94.0)^a^	97.8	1.3	1.0	89.9
16	n-Propanol	12	80.5(83.1)^a^	98.4	1.1	0.5	79.2
17	Ethanol	12	69.8(70.1)^a^	98.3	1.0	0.7	68.6
18	Methanol	12	2.2	91.9	7.3	0.8	2.0
19	—	12	95.0	77.68	21.9	0.4	73.8
20	Water	12	99.3	74.42	25.4	0.2	73.9

Reaction conditions: temperature = 35°C; pressure = 101.325 kPa; magnetic stirring = 650 r/min; air flow rate = 10 ml/min; reactant/solvent = 10:1 (w/w).

a : conversion at 18th hour.

Based on the data presented above, it can be confirmed that despite aprotic solvents giving higher reaction rates than protic solvents, it is unfavorable for the 2-ETH oxidation. Compared with organic solvent-free conditions (only water was used), the selectivity of 2-ETA obtained in aprotic solvents decreased obviously. Oppositely, the protic solvents with suitable polarity significantly improve the selectivity of 2-ETA.

We speculate that the different product distributions are mainly due to the intermolecular forces generated between the solvent molecules and reactants. The relatively weak van der Waals forces and dipole interactions in aprotic solvents (n-hexane, c-hexane) have no significant effect on the reaction. However, for the protic solvents, the possible formation of hydrogen bonds in the oxidation system could slow down the reaction from 2-ETA to peroxy-acid. And the reaction rate continually decreases till inactive with the increase of polarity and the decrease of alcohol molecule size. Therefore, *i*propanol as the solvent presents the highest yield of 2-ETA in the autoxidation of 2-ETH.

### 3.2 Solvent Protection Phenomenon in *i*propanol

**FIGURE 1:**
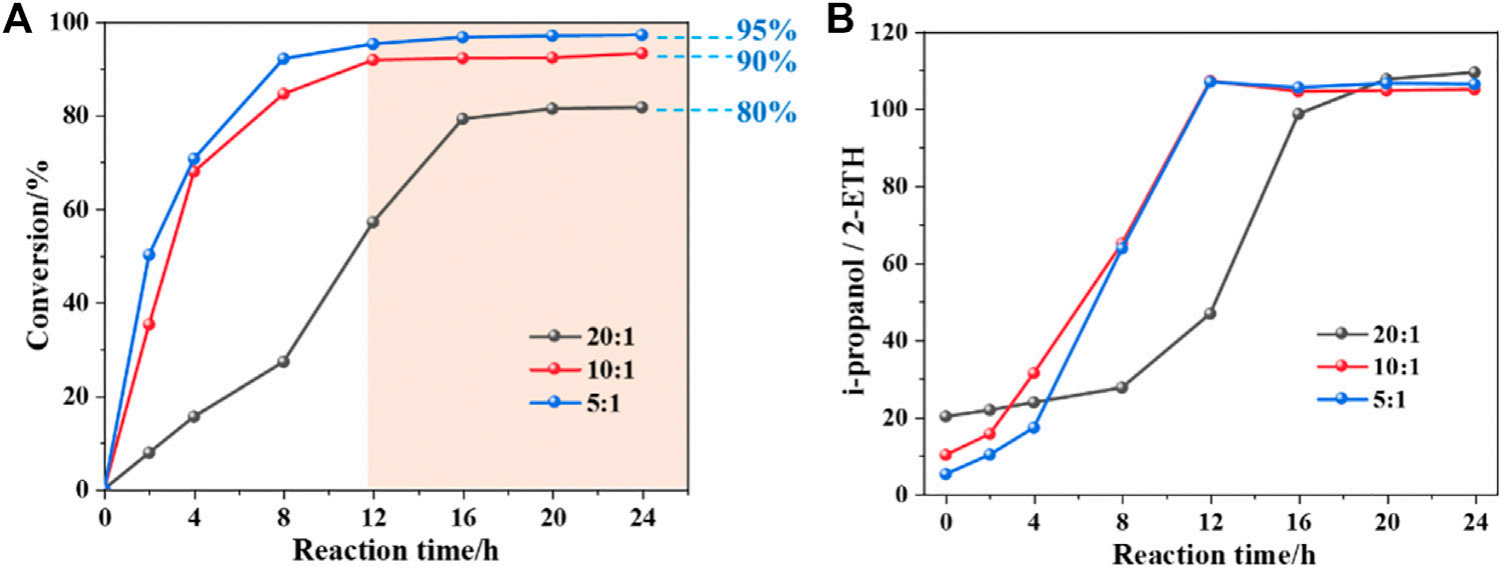
The protection phenomenon in *i*propanol: **(A)** the conversion of 2-ETH as a function of time and **(B)** the *i*propanol/2-ETH in different 2-ETH mass ratios as a function of time.

In the protic solvents environment (*i*propanol), the conversion of 2-ETH cannot be continuously increased with the extension of the reaction time. In Figure 1A, when the mass of *i*propanol decreases, the reaction rate gradually rises. After 16 h, the conversion of 2-ETH with different *i*propanol/2-ETH mass ratios (20:1, 10:1 and 5:1) tend to be flat and stabilized at around 80%, 90%, and 95%, respectively. It can be inferred that when a mass of protic solvent molecules enclose 2-ETH, strong intermolecular forces (hydrogen bonds) are probably generated, and the impact of such intermolecular forces becomes more significant with increasing *i*propanol/2-ETH ratio. The unconverted reactant is then “protected” through the interaction, thereby inhibiting the further occurrence of oxidation. Interestingly, a constant *i*propanol/2-ETH ratio of 105/1 (mass) was found in the final composition of the reacted mixture regardless of their original *i*propanol/2-ETH ratio in the feed (Figure 1B). It might be due to the relative “protection” effect or even a thermodynamic equilibrium of oxidation system.

### 3.3 Regulation of Intermolecular Forces

#### 3.3.1 Mixed Solvent Environment

The protic solvent (methanol, M; ethanol, E, and *i*propanol, *i*P) and aprotic solvent (n-hexane, n-H; c-hexane, c-H) are mixed for the reaction. During the oxidation (Table 3), the B-V reaction has strong protic solvent sensitivity. In the presence of a small amount of protic solvent, the selectivity of 2-ETA shows a substantial increase from 66%∼74% to 86% ∼93%. And the conversions remain at a high level (>75%), which are also much higher than those obtained in single-component protic solvents.

**TABLE 3:** Product distribution after 2 h in mixed solvent conditions.

Entry	Solvent	Conversion/%	Selectivity/%			Yield/%
			2-ETA	i-Heptyl formate	Others	
21	n-H+M	93.6	91.2	8.5	0.3	85.3
22	n-H+E	86.0	86.3	13.5	0.2	74.2
23	n-H+*i*P	87.9	90.9	8.8	0.3	79.9
24	c-H+M	86.7	92.8	6.8	0.4	80.5
25	c-H+E	84.8	92.1	7.5	0.3	78.1
26	c-H+*i*P	82.6	92.4	7.3	0.3	76.3

Reaction conditions: temperature = 35°C; pressure = 101.325 kPa; magnetic stirring = 650 r/min; air flow rate = 10 ml/min; reactant/solvent = 10:1 (w/w). n-H: n-hexane; c-H: c-hexane.

When the reaction proceeds (Table 4), the time to the reaction endpoint is significantly shortened compared to the single-component solvent condition. Though the protic solvent (methanol) has obviously limitation for oxidation, the abundant aprotic solvent will weaken the effect. The two types of solvents exert a synergistic effect with aprotic/protic = 29/1 (w/w). In the mixed solvent (entries 27, 31), the yield reaches ∼90%. However, due to the presence of the protic solvent, the hydrogen bond effect still exists, which could limit the further conversion.

**TABLE 4:** Product distribution of reaction endpoint in mixed solvent conditions.

Entry	Reaction time/h	Solvent	Conversion/%	Selectivity/%			Yield/%
				2-ETA	i-Heptyl formate	Others	
27	4	n-H+M	97.9	89.0	10.6	0.4	87.2
28	8	n-H+E	98.4	86.9	12.9	0.2	85.5
29	12	n-H+*i*P	96.9	88.6	11.2	0.2	85.9
30	4	c-H+M	96.7	91.7	7.8	0.5	88.7
31	8	c-H+E	97.6	94.0	5.6	0.4	91.8
32	12	c-H+*i*P	96.3	89.1	10.6	0.3	85.8

Reaction conditions: temperature = 35°C; pressure = 101.325 kPa; magnetic stirring = 650 r/min; air flow rate = 10 ml/min; reactant/solvent = 10:1 (w/w). n-H: n-hexane; c-H: c-hexane.

#### 3.3.2 The Mass Ratio of *i*propanol to Reactant

**FIGURE 2:**
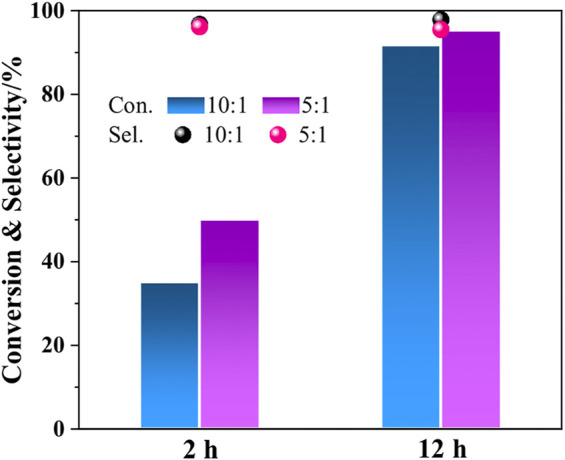
Evaluation results of adjusting the mass ratio of 2-ETH.

The influence of ratio of reactant to *i*propanol is further studied. As shown in Figure 2, the conversion of 2-ETH is improved from 35.2% to 50.2% *via* increasing the mass ratio of 2-ETH/*i*propanol from 10:1 to 5:1. Obviously, the protection effect of the solvent is reduced. After 12 h, the 2-ETH conversion with the mass ratio of 5:1 reached 95.5%. However, the selectivity of 2-ETA decreased slightly (from 96.2% to 95.5%). This could imply that, for the rearrangement step, the promotion of *i*propanol on hydrogen transfer is receded. The hydrogen bond effect could decrease with the higher 2-ETH/*i*propanol mass ratio (5:1).

#### 3.3.3 Influence of Evaporating Protic Solvent

**FIGURE 3:**
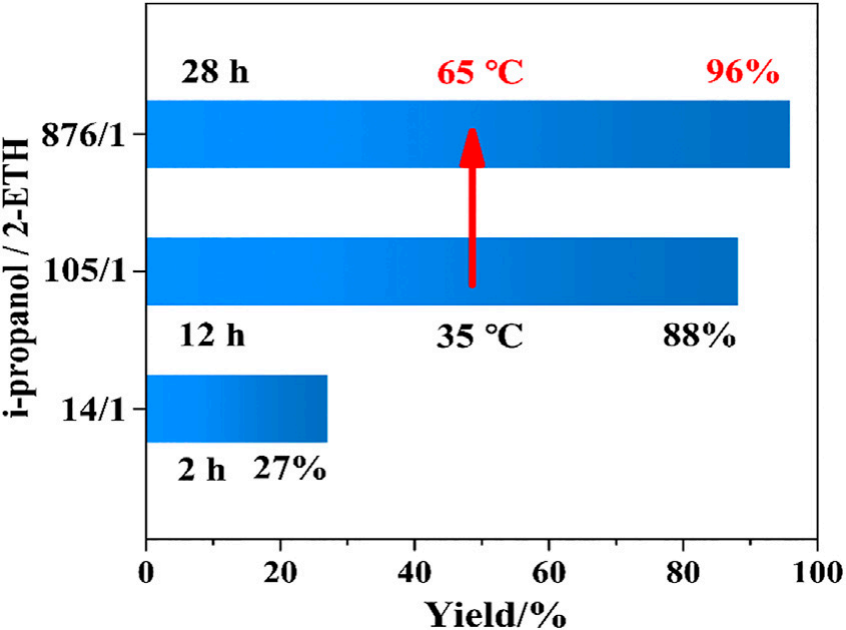
Evaluation results before and after solvent evaporation method.

As described above, the speculated hydrogen bonds between *i*propanol and 2-ETH inhibit the oxidation of 2-ETH and the 2-ETA yield is only 88.2% (Figure 3). To accelerate the oxidation, the solvent evaporation was implemented. Specifically, when the isothermal oxidation cannot proceed further, i.e., the conversion stalls, we raise the temperature from 35 to 65°C in order to evaporate the solvent, and a superior yield of 2-ETA (96.0%) was finally achieved after 28 h of processing. During the evaporation, the *i*propanol surrounding the 2-ETH molecules was gradually removed. Therefore, the strong intermolecular forces in the reaction system are weakened, and the previously protected 2-ETH molecules will continue to undergo the autoxidation. Apparently, the higher temperature will accelerate thermal movement of molecules and promote the conversion rate. We have performed the reaction at 55°C in *i*propanol, and a relatively higher conversion of about 94.4% but much lower 2-ETA selectivity of 90.8% were obtained (Supplementary Table S1). The higher mass ratio of 2-ETH in the reaction system given the faster conversion but the selectivity dropped down. For the solvent-free condition, a lower selectivity (77%) was obtained. It further demonstrated that conducting the reaction at higher temperature or 2-ETH concentration directly was not favorable for the selective synthesis of 2-ETA. However, in the evaporation method, removing the protic solvent *via* raising temperature at the equilibrium state will significantly enhance the oxidation of 2-ETH. It ensured both higher conversion and selectivity. Therefore, the presence of protic solvent and suitable temperature are the dominant factors for the formation of 2-ETA. The state-of-the-art results of the oxidation of 2-ETH and our results are compared in Supplementary Table S2. Obviously, the solvent evaporation method has many advantages: 1) mild reaction condition (35°C, 101.325 kPa); 2) no alkaline substances; 3) catalyst-free. With the high 2-ETA yield, it shows excellent industrial application prospects.

### 3.4 The Specific Manifestation of the Solvent Effect

#### 3.4.1 The Interaction Analysis Between *i*propanol and 2-ETH

**FIGURE 4:**
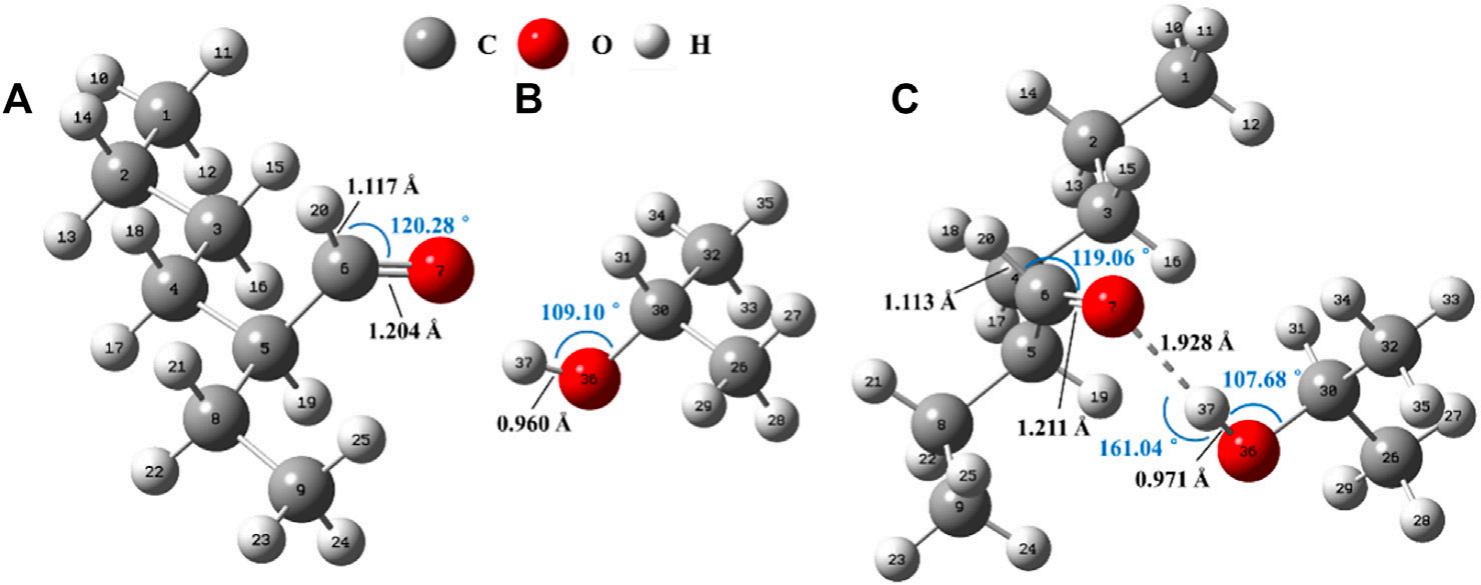
Optimized structures of **(A)** isolated 2-ETH, **(B)** isolated *i*propanol and **(C)** associated molecules.

We first use *i*propanol/2-ETH system as an example to specifically explain the computational model. The *i*propanol will enclose the 2-ETH molecule after it enters the continuous solvent field and constructs the associated state (Figure 4). Under the influence of one *i*propanol molecule, the C6 = O7 bond increases by 0.007 Å and C6-H20 bond shortens by 0.004 Å compared with the unassociated one. The distance between O7 and H37 is 1.928 Å, smaller than the sum of the van der Waals radii of the two atoms (2.5 Å) and within 1.5–2.2 Å. The O7-H37-O36 bond angle is 161.035°, between 130° and 180°, and close to a straight line. Such distance and angle indicate the formation of hydrogen bond between the two molecules. From the natural bond orbital (NBO) analysis (Supplementary Table S3) (Glendening et al., 2001), the donor-acceptor interaction between the O7 lone-pair electron and the anti-bonding orbital of -O36H37 group is significant (E^(2)^ = 30.56 kJ mol^−1^). And the intermolecular electrons accumulate towards O7 and O36 consequently (Supplementary Figure S1). The 2-ETH molecule becomes more stable in *i*propanol and the association energy (ΔE_a_) is −47.21 kJ/mol (ΔE_a_ = ΔE_Electronic energy-ZPE correction_ = E_associated_-E_2-ETH_-E_*i*propanol_, Supplementary Table S4). As a result, its oxidation rate will decrease.

#### 3.4.2 Molecular Structure and Energy Change

**FIGURE 5:**
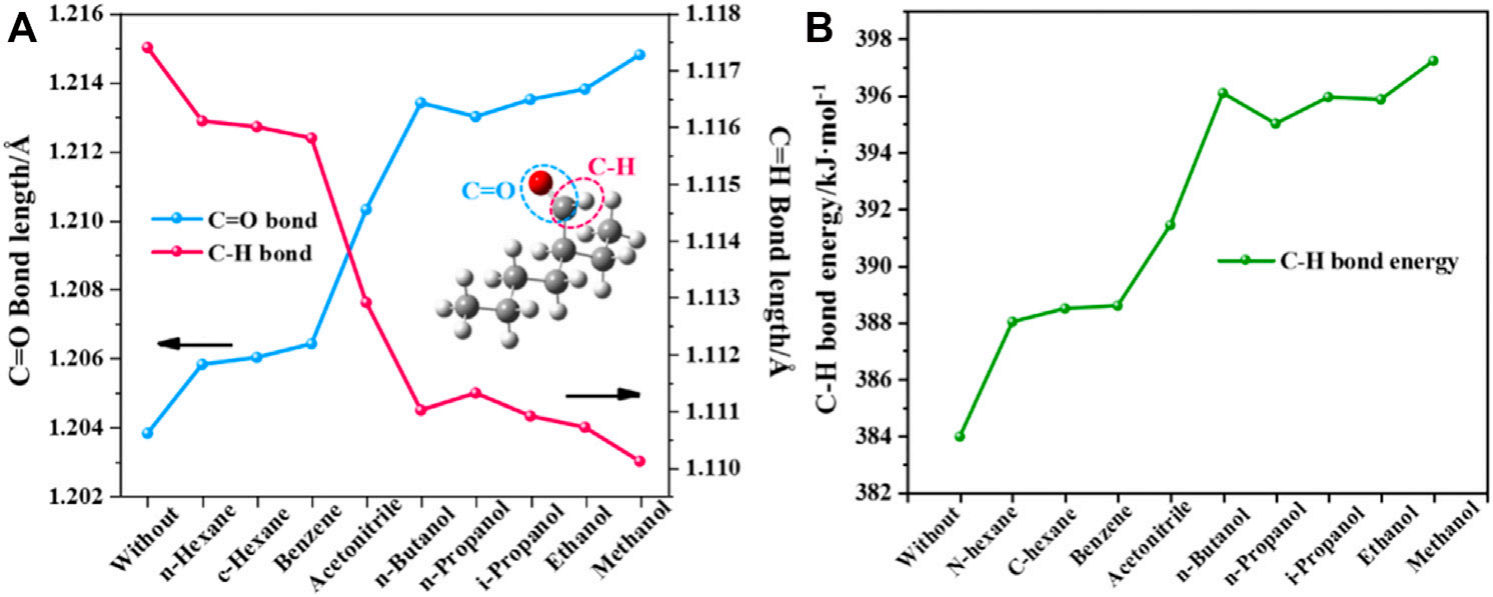
Calculated **(A)** Molecule structure parameters and **(B)** C-H bond energy change of 2-ETH in different solvents.

To further understand the specific effect of the solvent on 2-ETH, the molecular structures were optimized in different solvents by using the SMD solvation model (Figure 5A). Compared with the solvent-free condition, the C=O bond length of 2-ETH gradually increases, suggesting that the increasing solvent polarity enhances the intermolecular force by forming hydrogen bond with the solvent molecule. In contrast, the C-H bond length continually decreases. As shown in Figure 5B, the C-H bond energies in solvents are all higher than that in vacuum, and it increases significantly (>14 kJ/mol) in the protic solvents. These results indicate that the addition of protic solvent will increase the difficulty of C-H bond breaking and thus inhibit the oxidation progress.

#### 3.4.3 Frequency Analysis of Aldehyde Groups From Calculation

**FIGURE 6:**
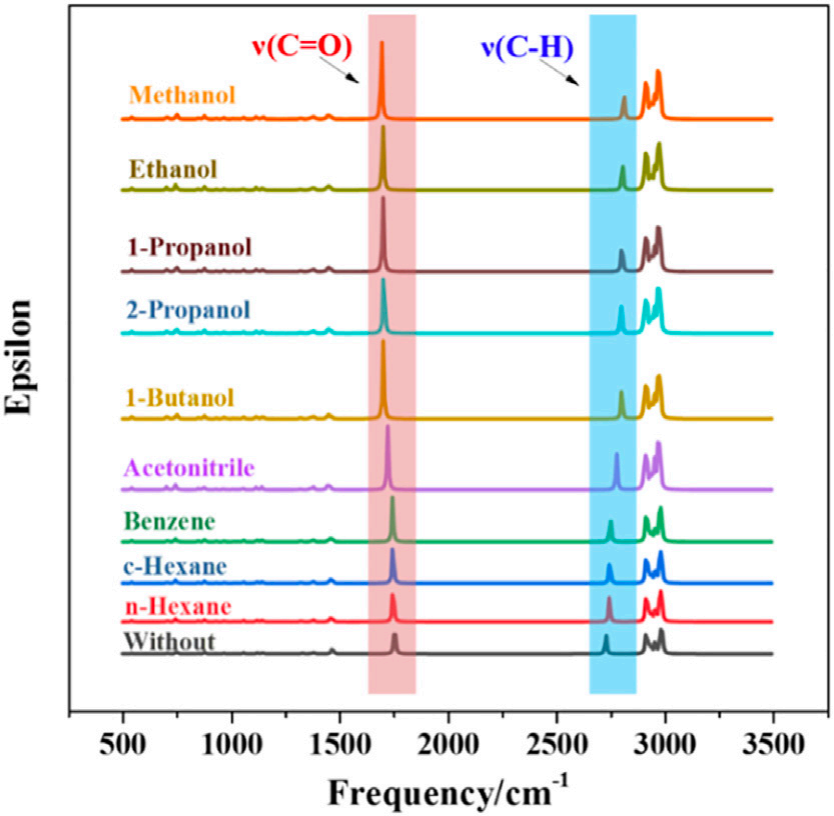
Calculated infrared spectra of 2-Ethylhexanal in different solvents.

Solvent effect will influence the vibration frequencies of ν_cal_(C=O) and ν_cal_(C-H) in aldehyde groups [Figure 6, scaling factor: 0.9640 (Kashinski et al., 2017)]. The deviation of the vibration frequency reflects the impact of different intermolecular forces. In aprotic solvents, the van der Waals force has little effect on the C=O bond. The ν_cal_(C=O) in n-hexane is only 7.58 cm^−1^ red shifted to the low wavenumbers. In protic solvents, the hydrogen bond (O-H…O) could form between the -OH (solvent) and C=O (2-ETH) groups, and the red-shift of ν_cal_(C=O) is more apparent (>50 cm^−1^). For ν_cal_(C-H), however, the blue-shift becomes more significant with the increase of solvent polarity. And the C-H bond becomes stronger accordingly to slow down the oxidation.

#### 3.4.4 The Parameter Equation Analysis of Solvent Effect

To elaborate the internal connection of the solvent effect on 2-ETH molecules and oxidation reactivity, the parameter equations are introduced (Brownstein, 1960; Gutmann, 1967; Reichardt, 1979; Kamlet et al., 1983; Swain et al., 1983; Redondo et al., 1986). The parameter values are summarized in Supplementary Tables S5, S6. As shown in Supplementary Figure S2, the main factor of the change of ν_cal_(C=O) is the electrophilic ability (AN value) and solvent polarity (S value). The solvent will be easier to accept electrons with the increased AN value, which will form the supply-receive effect of electron pair between the solvent molecular orbitals and lone-pair electron. For LSER (Supplementary Table S7), comparing the absolute values of α and β coefficient (39.74 > 1.12), it is implied that the solvent is mainly used as a hydrogen bond donor. The O of C=O bond provides a lone-pair electron, and the hydroxyl group of the alcohol solvent delivers the proton.

**FIGURE 7:**
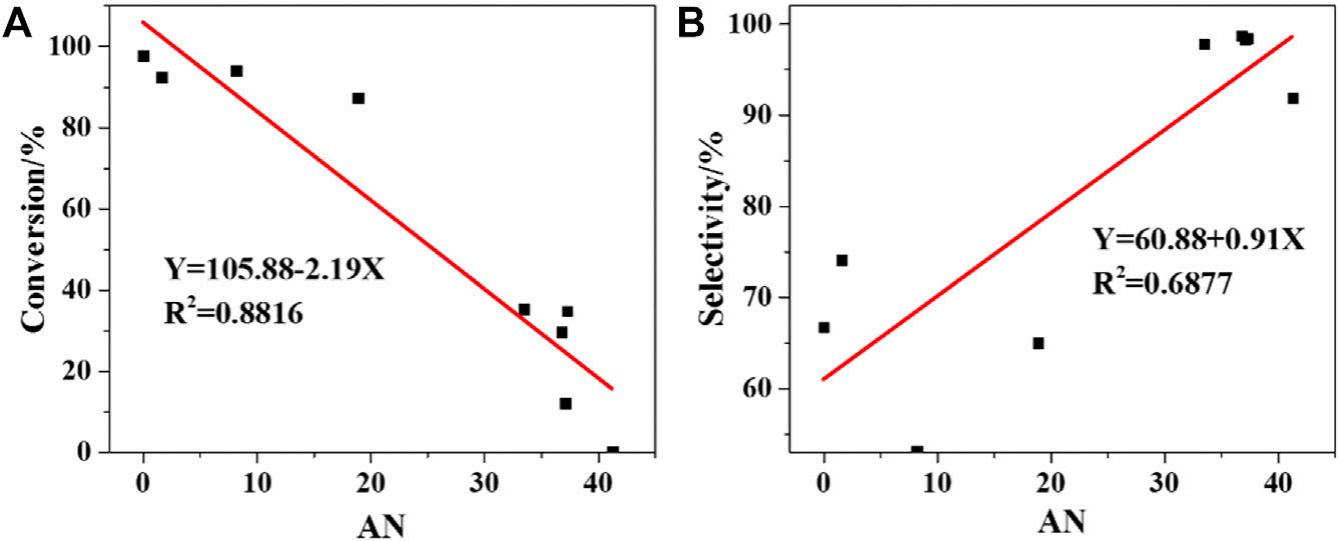
Correlation between AN and reactivity: **(A)** Conversion of 2nd hour, **(B)** Selectivity.

In Figure 7, the 2-ETH conversion drops down with the increased solvent electrophilcity, resulting in a lower oxidation rate. In addition, solvent electrophilicity has a more prominent influence on 2-ETH conversion (*R* ^2^ = 0.8816) than 2-ETA selectivity (*R* ^2^ = 0.6877).

The 2-ETH conversion and 2-ETA selectivity are correlated through the multi-parameter LSER equation (Table 5). For selectivity, the coefficients of α and β are positive, which indicates that the hydrogen bond effect is favorable to 2-ETA selectivity. Meanwhile, the coefficient of α (20.62) is greater than that of β (11.25) and so indicated that the solvent provides protons to form the hydrogen bond with the solute thus improving 2-ETA selectivity. It is suggested that in the B-V oxidation rearrangement reaction, the solute-solvent hydrogen bond is beneficial to the occurrence of H transfer, thereby increasing the selectivity of the 2-ETA.

**TABLE 5:** The correlation between LSER equations and conversion and selectivity.

Entry	Multiple regression expression	*R* ^2^
Conversion	C = 98.02 + (–56.37π* + 14.30δ) –189.60α + 199.07β	0.8985
Selectivity	S = 69.78 + (–26.91π* – 3.78δ) + 20.62α + 11.25β	0.9612

## 4 Conclusion

In this study, the solvent effect in the oxidation of 2-ETH to 2-ETA by air was studied in detail by combining both experimental and computational methods. The reaction exhibits distinct reactivity and product selectivity under various solvent conditions. As the polarity of the solvent increases, the intermolecular force changes from weak van der Waals force to more substantial hydrogen bond. The selectivity of 2-ETA is higher in alcohol solvents, and the best single-component solvent is *i*propanol. Due to the strong solvent-reactant interaction, 2-ETH is protected, and the oxidation exists a limitation on a particular mass ratio (105/1). Based on this nature, the solvent evaporation method is proposed. With the intermolecular force gradually being removed, the 2-ETH is continually oxidized to 2-ETA and finally achieves the highest yield of 96%. Moreover, theoretical calculations confirm that intermolecular forces are the dominant factor of the solvent effect. It will influence the structure of 2-ETH molecule and stabilize the C-H bond. It is evident that the 2-ETH conversion decline when the hydrogen bond is formed. Meanwhile, the hydrogen bond effect is positively correlated to the selectivity of 2-ETA, which suggests that H transfer in the B-V reaction is promoted and 2-ETA is the most favorable product. The novel method and theoretical analysis provide a valuable application prospect in the aldehyde oxidation industry.

## Data Availability

The original contributions presented in the study are included in the article/Supplementary Material, further inquiries can be directed to the corresponding authors.
