# Supplementary material for: Solvent Effect on Product Distribution in the Aerobic Autoxidation of 2-Ethylhexanal: Critical Role of Polarity
Source: Front Chem. 2022 Mar 25;10:855843. doi: 10.3389/fchem.2022.855843 (PMC8989829; doi:10.3389/fchem.2022.855843)
Supplement: Supplementary file 1 [file DataSheet1.docx]

**Supplementary Material**

**Origin of solvent effect on product distribution in the aerobic autoxidation of 2-Ethylhexanal: critical role of hydrogen bonds**

**Zheng Wang^1,^†, Yitong Qin ^1,^†, Huijiang Huang^1^, Guobing Li^1^, Yan Xu ^1^, Peng Jin^2,^*, Bo Peng^3,^*, Yujun Zhao ^1,^***

^1^Key Laboratory for Green Chemical Technology of Ministry of Education, School of Chemical Engineering and Technology, Tianjin University, Tianjin 300072, China.

^2^School of Materials Science and Engineering, Hebei University of Technology, Tianjin, 300130, China

^3^SINOPEC Research Institute of Petroleum Processing, Beijing 100083, China

*** Correspondence:**Peng Jin
pengjin@hebut.edu.cn

Bo Peng
pengbo.ripp@sinopec.com

Yujun Zhao
yujunzhao@tju.edu.cn

†These authors have contributed equally to this work


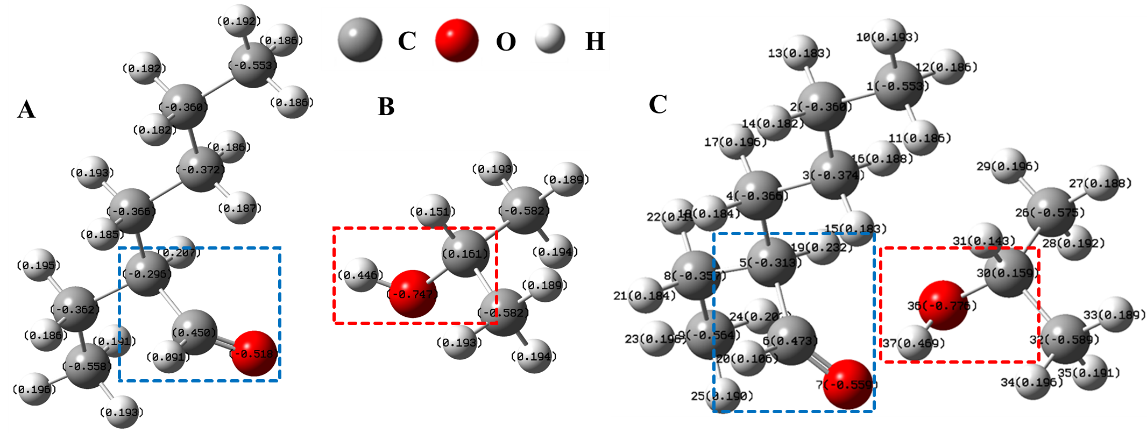


**FIGURE S1** | NBO charge distribution of (A) 2-ETH, (B) i-propanol and (C) associated molecules.


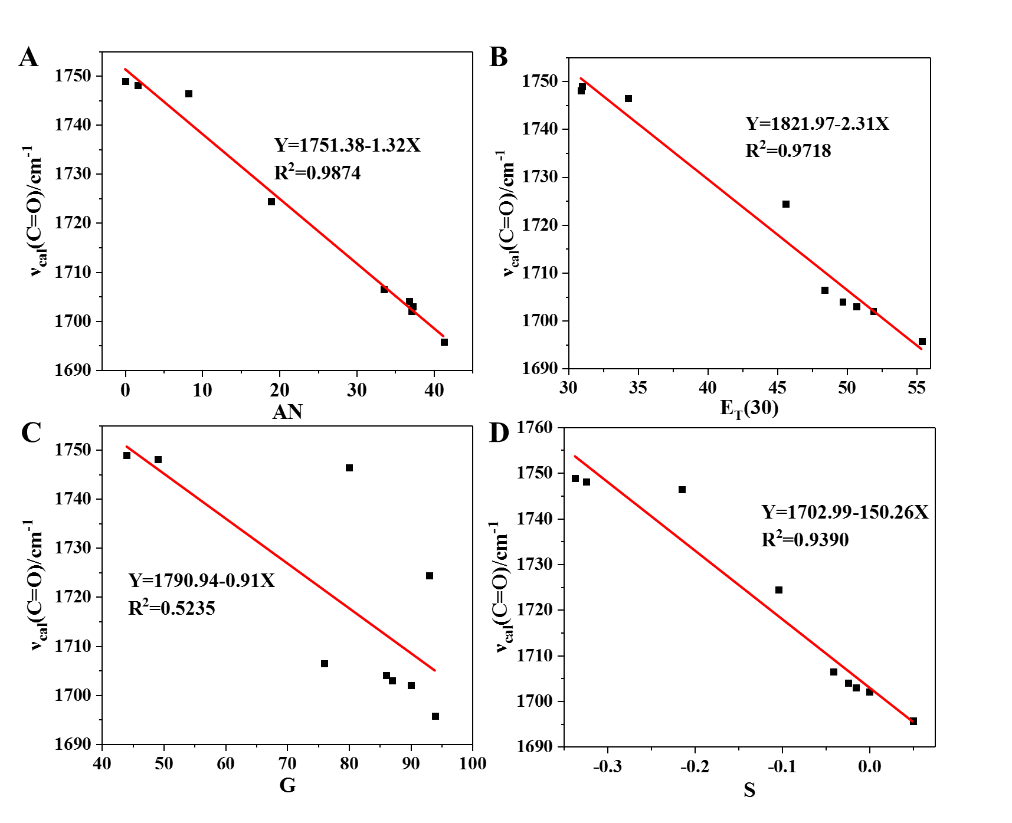


**FIGURE S2** | The correlation between single parameter equations and ν_cal_(C=O): (A) AN, (B) E_T_(30), (C) G, (D) S.

The linear correlation formula of the single parameter equation is as follows (1), where ν represents ν(C=O) in solvents, ν_0_ represents ν(C=O) in solvent-free, X represents the solvent parameter, and k represents sensitivity to this parameter.

$\nu\text{=}\nu_{0}\text{+k}X$ (1)

The slopes of all single-parameter equations are all negative, indicating that ν_cal_(C=O) is negatively correlated with solvent parameters. The solvent acceptance number (AN) is used to reflect the solvent electrophilicity (Lewis acidity). The G value is mainly used to reflect the non-specific interaction. The S value is used to reflect the polarity of the solvent. The fitting value (1821.97 cm^-1^) of E_T_(30) equation is entirely deviated from the ν_cal_(C=O) (1756.48 cm^-1^) under solvent-free condition. The lower R^2^ (0.5235) of G is provided that the non-specific interaction is not the main factor affecting the vibration frequency of the C=O.

**TABLE S1 |** The effect of reaction temperature on oxidation reaction.

| Temperature/°C | Conversion/% | Selectivity/% | | | Yield/% |
| --- | --- | --- | --- | --- | --- |
|  |  | 2-ETA | i-Heptyl formate | Others |  |
| 15 | 61.8 | 95.5 | 1.7 | 2.9 | 59.0 |
| 35 | 91.9 | 97.8 | 1.3 | 0.9 | 89.9 |
| 55 | 94.4 | 90.8 | 1.8 | 7.3 | 85.8 |

Reaction conditions: pressure = 101.325 kPa; magnetic stirring = 650 r/min; air flow rate = 10 mL/min; reactant / solvent = 10:1 (w/w), reaction time = 12h.

**TABLE S2 |** Summary of research results on the oxidation of 2-Ethylhexanal.

| Entry | Temp.(°C) | Oxidation  conditions | Alkaline  addition | Yield(%) | Solvent | Cat. | Ref. |
| --- | --- | --- | --- | --- | --- | --- | --- |
| 1 | Room | O_2_ bubble | - | 8 | Methanol | - | (Lehtinen and Brunow, 2000) |
| 2 | Room | O_2_ | - | 83 | n-Heptane | Mn(II) | (Lehtinen and Brunow, 2000) |
| 3 | Room | m-CPBA | - | 91 | *i*Propanol | - | (Lehtinen et al., 2001) |
| 4 | Room | O_2_ | - | 88 | Water | - | (Shapiro and Vigalok, 2008) |
| 5 | 25 | Air 30 bar | - | 85 | Heptane | - | (Vanoye et al., 2013b) |
| 6 | Room | O_2_ 5 bar | - | 75 | Heptane | - | (Vanoye et al., 2013a) |
| 7 | Room | O_2_ 5 bar | Na^+^ | 91 | Heptane |  | (Vanoye et al., 2014) |
| 8 | Room | O_2_ 5 bar | Na^+^ | 95 | Heptane | Mn(II) | (Vanoye et al., 2014) |
| 9 | 25 | O_2_ 7.5 bar | Na^+^ | 97 | - | Mn(II) | (Vanoye et al., 2016) |
| 10 | 50 | O_2_ | NaOH | 99 | Water | Ag_2_O/IPr | (Liu et al., 2015) |
| 11 | 50 | O_2_ | NaOH | 99 | Water | Cu(II) | (Liu and Li, 2016) |
| 12 | 50 | O_2_ | Na_2_CO_3_ | 99 | Water | Fe^III^Mo_6_ | (Yu et al., 2017) |
| 13 | 30 | O_2_ | - | 90 | Acetonitrile | NHPI | (Vanoye et al., 2019) |
| 14 | 30 | O_2_ | - | 92 | Water | NHPI | (Vanoye et al., 2019) |
| 15 | 35 | Air bubble | - | 96 | *i*Propanol | - | This work |

**TABLE S3 |** Second order perturbation theory analysis of Fock matrix in NBO.

| Donor NBO (i) | Acceptor NBO (j) | E(j)-E(i)/a.u. | F(i,j)/a.u. | E^(2)^/kJ·mol^-1^ |
| --- | --- | --- | --- | --- |
| LP(1)O7 | BD^*^(1)O36-H37 | 1.16 | 0.045 | 8.9996 |
| LP(2)O7 | BD^*^(1)O36-H37 | 0.75 | 0.068 | 30.5567 |

LP: lone-pair electron; BD^*^: anti-bond orbital.

**TABLE S4 |** Thermochemistry of associated molecules and unassociated molecules.

| Entry | Thermochemistry/a.u. | |
| --- | --- | --- |
|  | Electronic energy with zero-point energy correction | Gibbs free energy |
| 2-ETH | -389.611 | -389.652 |
| i-Propanol | -194.305 | -194.333 |
| Associated | -583.933 | -583.986 |
| ΔE | -0.018 | -0.002 |

**TABLE S5 |** Single parameter values of different solvents.

| Solvent | AN | E_T_(30) | G | S |
| --- | --- | --- | --- | --- |
| n-Hexane | 0.00 | 31.00 | 44.00 | -0.34 |
| c-Hexane | 1.60 | 30.90 | 49.00 | -0.32 |
| Benzene | 8.20 | 34.30 | 80.00 | -0.22 |
| Acetonitrile | 18.90 | 45.60 | 93.00 | -0.10 |
| n-Butanol | 36.80 | 49.70 | 86.00 | -0.02 |
| *i*Propanol | 33.50 | 48.40 | 76.00 | -0.04 |
| n-Propanol | 37.30 | 50.70 | 87.00 | -0.02 |
| Ethanol | 37.10 | 51.90 | 90.00 | 0.00 |
| Methanol | 41.30 | 55.40 | 94.00 | 0.05 |

**TABLE S6 |** Swain and LSER parameter values of different solvents.

| Solvent | Swain | | LSER | | | |
| --- | --- | --- | --- | --- | --- | --- |
|  | A_j_ | B_j_ | π* | δ | α | β |
| n-Hexane | 0.01 | -0.01 | -0.04 | 0 | 0.00 | 0.00 |
| c-Hexane | 0.02 | 0.06 | 0.00 | 0 | 0.00 | 0.00 |
| Benzene | 0.15 | 0.59 | 0.59 | 1 | 0.00 | 0.10 |
| Acetonitrile | 0.37 | 0.86 | 0.75 | 0 | 0.19 | 0.40 |
| n-Butanol | 0.61 | 0.43 | 0.47 | 0 | 0.84 | 0.84 |
| *i*Propanol | 0.59 | 0.44 | 0.48 | 0 | 0.76 | 0.84 |
| n-Propanol | 0.63 | 0.44 | 0.52 | 0 | 0.84 | 0.90 |
| Ethanol | 0.66 | 0.45 | 0.54 | 0 | 0.86 | 0.75 |
| Methanol | 0.75 | 0.50 | 0.60 | 0 | 0.98 | 0.66 |

**TABLE S7 |** The correlation between Swain and LSER equations and ν_cal_(C=O).

| Equation | Multiple regression equation | R^2^ |
| --- | --- | --- |
| Swain | ν_cal_(C=O) = 1750.18 –79.35A _j_ + 7.46B_j_ | 0.9911 |
| LSER | ν_cal_(C=O) = 1748.07 + (–20.93π* + 10.78δ) –39.74α –1.12β | 0.9998 |

The Swain equation is used to describe the specific interaction in solvent. The A_j_ represents the dissolution tendency of anions (Lewis acid), and B_j_ reflects the dissolution tendency of cations (Lewis basic). The A_j_ has a negative correlation with ν_cal_(C=O), which conforms to the trend of red-shift.

The linear solvation energy relationship (LSER) can explain the solute-solvent interaction. The π* is the dipole/polarizability index and δ is the correction of π*. α represents the solvent HBD (hydrogen bond donor) acidity, which describes the ability of the solvent to provide protons to the solute when forming a hydrogen bond with the solute. β stands for solvent HBA (hydrogen bond acceptor) alkalinity, which reflects the ability of the solvent to accept solute protons and provide electron pairs when forming hydrogen bonds with the solute. The π* coefficient is negative, indicating that the weak non-specific force in the solvent will cause the red-shift of ν_cal_(C=O).

**References**

Lehtinen, C., and Brunow, G. (2000). Factors affecting the selectivity of air oxidation of 2-ethyhexanal, an alpha-branched aliphatic aldehyde. *Org. Process Res. Dev.* 4**,** 544-549. doi: 10.1021/op000045k

Lehtinen, C., Nevalainen, V., and Brunow, G. (2001). Experimental and computational studies on solvent effects in reactions of peracid-aldehyde adducts. *Tetrahedron* 57**,** 4741-4751. doi: 10.1016/S0040-4020(01)00397-0

Liu, M., and Li, C.-J. (2016). Catalytic fehling's reaction: an efficient aerobic oxidation of aldehyde catalyzed by copper in water. *Angew. Chem. Int. Ed.* 55**,** 10806-10810. doi: 10.1002/anie.201604847

Liu, M.X., Wang, H.N., Zeng, H.Y., and Li, C.J. (2015). Silver(I) as a widely applicable, homogeneous catalyst for aerobic oxidation of aldehydes toward carboxylic acids in water-"silver mirror": From stoichiometric to catalytic. *Sci. Adv.* 1**,** e1500020. doi: 10.1126/sciadv.1500020

Shapiro, N., and Vigalok, A. (2008). Highly efficient organic reactions "on Water", "in Water", and both. *Angew. Chem. Int. Ed.* 47**,** 2849-2852. doi: 10.1002/anie.200705347

Vanoye, L., Abdelaal, M., Grundhauser, K., Guicheret, B., Fongarland, P., De Bellefon, C., et al. (2019). Reinvestigation of the organocatalyzed aerobic oxidation of aldehydes to acids. *Org. Lett.* 21**,** 10134-10138. doi: 10.1021/acs.orglett.9b04193

Vanoye, L., Aloui, A., Pablos, M., Philippe, R., Percheron, A., Favre-Réguillon, A., et al. (2013a). A safe and efficient flow oxidation of aldehydes with O_2_. *Org. Lett.* 15**,** 5978-5981. doi: 10.1021/ol401273k

Vanoye, L., Favre-Reguillon, A., Aloui, A., Philippe, R., and De Bellefon, C. (2013b). Insights in the aerobic oxidation of aldehydes. *Rsc Adv.* 3**,** 18931-18937. doi: 10.1039/c3ra42385a

Vanoye, L., Pablos, M., Smith, N., De Bellefon, C., and Favre-Reguillon, A. (2014). Aerobic oxidation of aldehydes: selectivity improvement using sequential pulse experimentation in continuous flow microreactor. *Rsc. Adv.* 4**,** 57159-57163. doi: 10.1039/c4ra12067a

Vanoye, L., Wang, J.D., Pablos, M., Philippe, R., De Bellefon, C., and Fayre-Reguillon, A. (2016). Continuous, fast, and safe aerobic oxidation of 2-ethyihexanal: pushing the limits of the simple tube reactor for a gas/liquid reaction. *Org. Process Res. Dev.* 20**,** 90-94. doi: 10.1021/acs.oprd.5b00359

Yu, H., Ru, S., Dai, G.Y., Zhai, Y.Y., Lin, H.L., Han, S., et al. (2017). An efficient iron(III)-catalyzed aerobic oxidation of aldehydes in water for the green preparation of carboxylic acids. *Angew. Chem. Int. Ed.* 56**,** 3867-3871. doi: 10.1002/anie.201612225
